# Beyond carbon capture: bioengineering cyanobacteria as solar-driven platforms for remediation of recalcitrant human-generated waste

**DOI:** 10.3389/fpls.2026.1899035

**Published:** 2026-07-20

**Authors:** Sandugash N. Seiilbek, Saima S. Mirza, Makpal M. Torekhanova, Nazgul A. Altybaeva, Raikhan K. Sydykbekova, Nurziya R. Akmukhanova, Barry D. Bruce

**Affiliations:** 1 Department of Biotechnology, Faculty of Biology and Biotechnology, Al-Farabi Kazakh National University, Almaty, Kazakhstan; 2 Department of Biochemistry, Cellular and Molecular Biology, University of Tennessee, Knoxville, TN, United States; 3 Department of Microbiology, University of Tennessee, Knoxville, TN, United States

**Keywords:** cyanobacteria biotechnology, environmental remediation, pesticide degradation, plastic pollution, wastewater treatment

## Abstract

Cyanobacteria are increasingly positioned as photosynthetically powered, genetically tractable chassis for next-generation environmental remediation-operating as living filters that couple solar energy capture to active detoxification and resource recovery. This review synthesizes current advances in cyanobacteria-based remediation of heavy metals, micro- and nanoplastics, pathogens, and persistent organic pollutants, with particular emphasis on metabolic mechanisms, bioengineering strategies, and practical environmental applications. This review outlines a bioengineering roadmap for deploying cyanobacteria in wastewater and impacted aquatic systems to sequester and reclaim toxic heavy metals, trap nano/microplastics, attenuate pathogenic microorganisms, and chemically degrade recalcitrant organic pollutants. In the field of metal capture, recent advances in “living materials” have enabled the embedding of cyanobacteria in regenerable matrices for efficient removal and subsequent reclamation. Mechanistic insights into species such as *Synechocystis* have clarified adsorption behavior and stress-response determinants for cadmium and related metals, defining tunable targets including transporters, exporters, and chelation modules for strain improvement. Cobalt and uranium handling can now be rationally engineered by rewiring metal homeostasis systems or exploiting high-capacity biosorption using scalable biomass platforms like *Spirulina*. Beyond metals, cyanobacterial extracellular polymeric substances (EPS) are being leveraged as engineered bio-based flocculants to remove polystyrene micro- and nanoplastics, while consortia-based designs are emerging to facilitate polymer transformation. Collectively, these advances motivate the development of modular, field-ready cyanobacterial platforms immobilized, sensor-guided, and biocontained that integrate pollutant capture and circular recovery within sustainable photobioremediation pipelines. However, significant challenges remain, including field-scale validation, environmental variability, biosafety considerations, biomass management, economic feasibility, and regulatory constraints. Addressing these limitations will be essential for the practical implementation of cyanobacterial remediation technologies.

## Graphical Abstract

**Figure:**
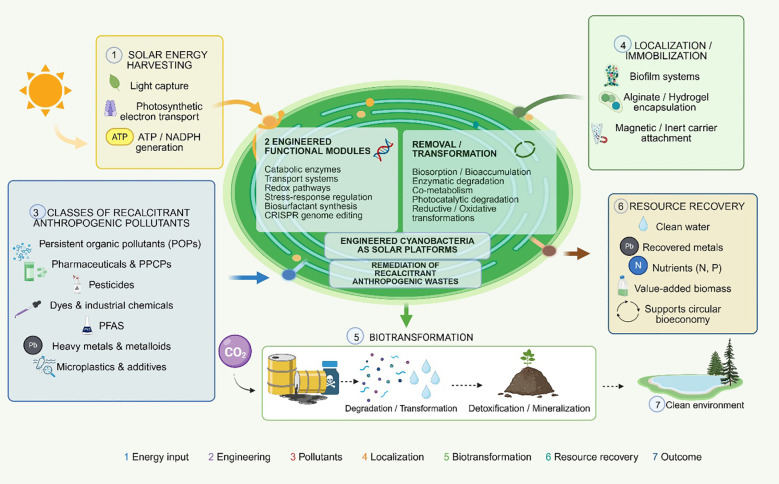

## 1 Introduction

Anthropogenic pollution of aquatic ecosystems has emerged as a critical global challenge in the Anthropocene epoch, characterized by the accelerating degradation of marine and freshwater environments (Häder et al., 2020; Quadra et al., 2019). Major pollution sources include sewage, excessive nutrient loading, crude oil, heavy metals, and plastics, which affect aquatic organisms even when pollution sources are distant from the impacted areas (Häder et al., 2020). Industrial activities, particularly mining, introduce toxic heavy metals into water bodies, while agricultural production and inefficient irrigation systems further contribute to ecosystem destabilization (Abenova et al., 2024). Emerging pollutants, including endocrine disruptors, semi-volatile organic compounds, and microplastics, pose unprecedented threats to aquatic biodiversity and water quality (Chawla et al., 2024; Madhav et al., 2020). Traditional restoration approaches such as dredging, phytoremediation, and ecological floating beds can reduce pollutant concentrations and improve dissolved oxygen levels, yet long-term ecosystem recovery requires integrated strategies that prioritize ecosystem functioning rather than solely water quality improvement (Häder et al., 2020; Chawla et al., 2024).

Water pollution threatens both human health and aquatic ecosystems due to the persistence and toxicity of pollutants. Major contaminants include heavy metals (Fe, Mn, Al, Cu, Zn, Pb, Cd, Cr, Ni, Hg), persistent organic pollutants (POPs), pesticides, pathogens, and emerging contaminants such as microplastics and pharmaceuticals (Rathi et al., 2021; Schwarzenbach et al., 2010; Morin-Crini et al., 2022). Conventional wastewater treatment plants (WWTPs) are often inadequate in removing these contaminants of emerging concern (CECs) due to their small size, chemical recalcitrance, and complex mixtures (Matesun et al., 2024). While advanced methods, including activated sludge systems, carbon-based filtration, and microbial fuel cells, show higher efficiency, they face limitations in sustainability, scalability, and energy demands (Sharma et al., 2019; Nishat et al., 2023). These challenges highlight the urgent need for innovative, solar-powered approaches to improve contaminant removal in aquatic environments.

**Figure 1:**
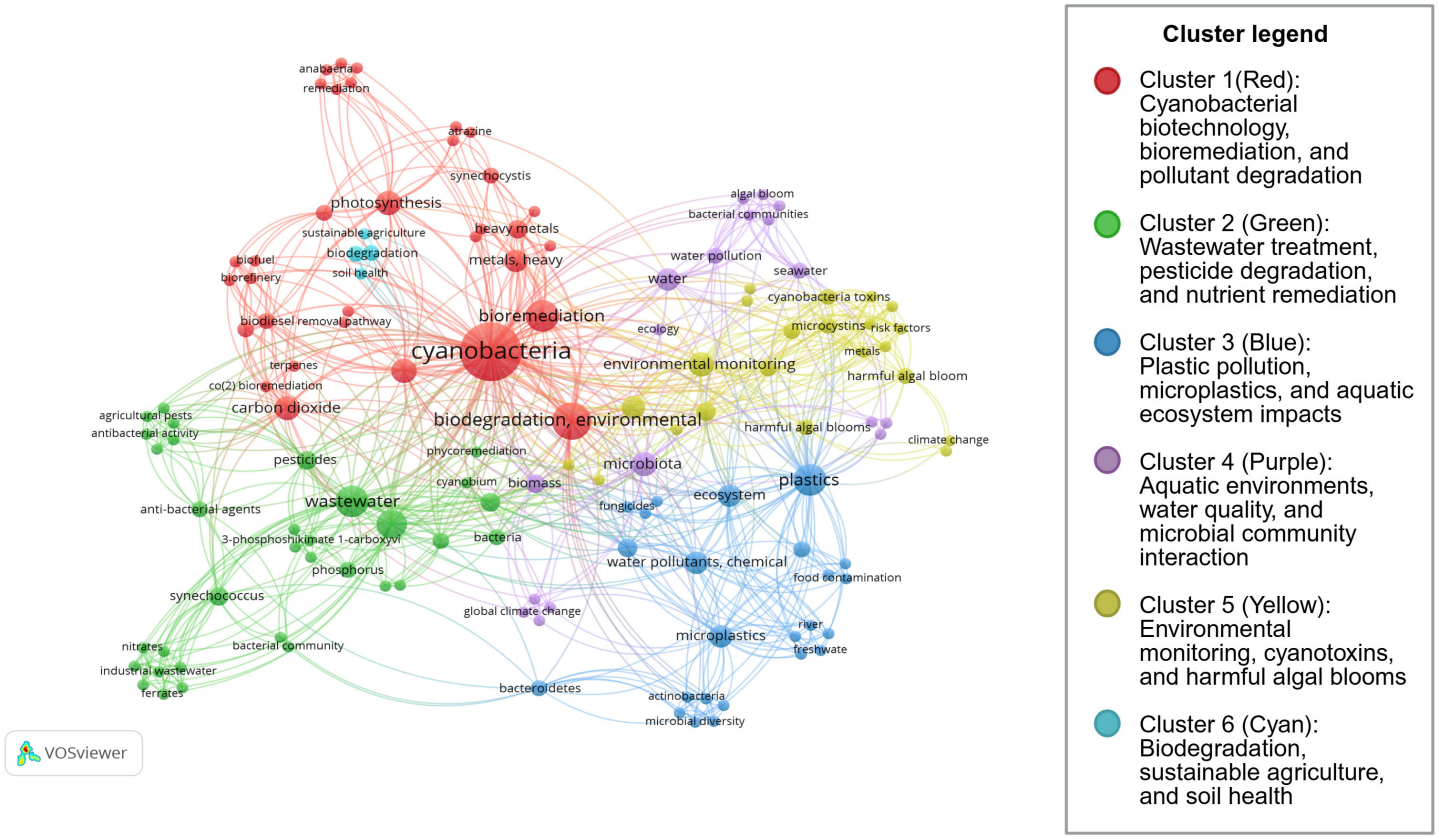
Keyword co-occurrence network generated using VOSviewer based on 2,179 PubMed publications (2016–2026). Colors represent thematic clusters, while node size and link strength indicate keyword frequency and co-occurrence relationships.

Beyond their well-documented role in carbon sequestration, cyanobacteria are emerging as versatile solar-driven platforms for the remediation of recalcitrant waste. These prokaryotic organisms perform oxygenic photosynthesis, require minimal growth inputs, and exhibit remarkable metabolic versatility (Lau et al., 2015; Cui et al., 2021). Several cyanobacterial genera, including *Anabaena*, *Microcystis*, *Nostoc*, and *Synechocystis*, have demonstrated the ability to tolerate, transform, or degrade synthetic pesticides, herbicides, and heavy metals (Kumar and Singh, 2017). Their potential applications in bioremediation are associated with phototrophic growth, nitrogen fixation, environmental adaptability, and flexible metabolic pathways. Furthermore, advances in genetic engineering and synthetic biology have enabled the development of cyanobacterial platforms with improved pollutant capture and transformation capabilities, although most applications remain at the laboratory or early development stages.To identify current research trends and major application areas of cyanobacteria in the remediation of persistent anthropogenic pollutants, a bibliometric keyword co-occurrence analysis was performed using VOSviewer (**Figure 1**). The analysis was based on 2,179 English-language publications retrieved from the PubMed database (2016–2026) using the search query *(cyanobacteria OR “blue-green algae”) AND (biotechnology OR bioremediation OR “wastewater treatment” OR pesticides OR “pesticide degradation” OR plastic OR microplastic OR “metal pollution”).* Keywords were analyzed using the full counting method with association-strength normalization.

The analysis revealed several major thematic clusters reflecting the structure of contemporary research in this field. These include: bioengineering and synthetic biology of cyanobacteria aimed at developing modified photosynthetic systems for enhanced pollutant transformation; bioremediation of heavy metals, encompassing processes such as biosorption, bioaccumulation, and detoxification of toxic metals; wastewater treatment and environmental remediation, where cyanobacteria are considered effective agents for the removal of nutrients and organic contaminants; biodegradation of organic pollutants and pesticides associated with anthropogenic pressure on agroecosystems; as well as the removal of micro- and nanoplastics, including processes mediated by EPS, which facilitate particle aggregation and binding. The identified clusters highlight the key directions of current research, positioning cyanobacteria as solar-driven photosynthetic platforms for the sustainable environmental remediation of recalcitrant anthropogenic pollutants.

The aim of this review is to provide a comprehensive overview of cyanobacteria-based strategies for the remediation of recalcitrant anthropogenic pollutants, with a focus on metabolic pathways, bioengineering approaches, and practical applications in freshwater and wastewater systems. By synthesizing recent advances and highlighting technical bottlenecks, this review seeks to inform the development of sustainable, scalable, and multifunctional cyanobacterial platforms for next-generation water remediation. Furthermore, the implementation of these strategies contributes to several United Nations Sustainable Development Goals (SDGs), including SDG 6 (Clean Water and Sanitation), SDG 7 (Affordable and Clean Energy), SDG 14 (Life Below Water), and SDG 15 (Life on Land), by improving water quality, enabling renewable bioenergy production, and preserving global ecosystems. Unlike previous reviews that primarily address cyanobacterial biotechnology, wastewater treatment, or synthetic biology separately, this review integrates these fields within the context of remediating recalcitrant anthropogenic pollutants. Particular emphasis is placed on the links between pollutant removal mechanisms, bioengineering strategies, practical implementation challenges, and opportunities for sustainable water remediation within a circular bioeconomy framework.

## 2 Cyanobacteria as solar-driven biotechnological platforms: from evolutionary adaptation to synthetic design

**Figure 2:**
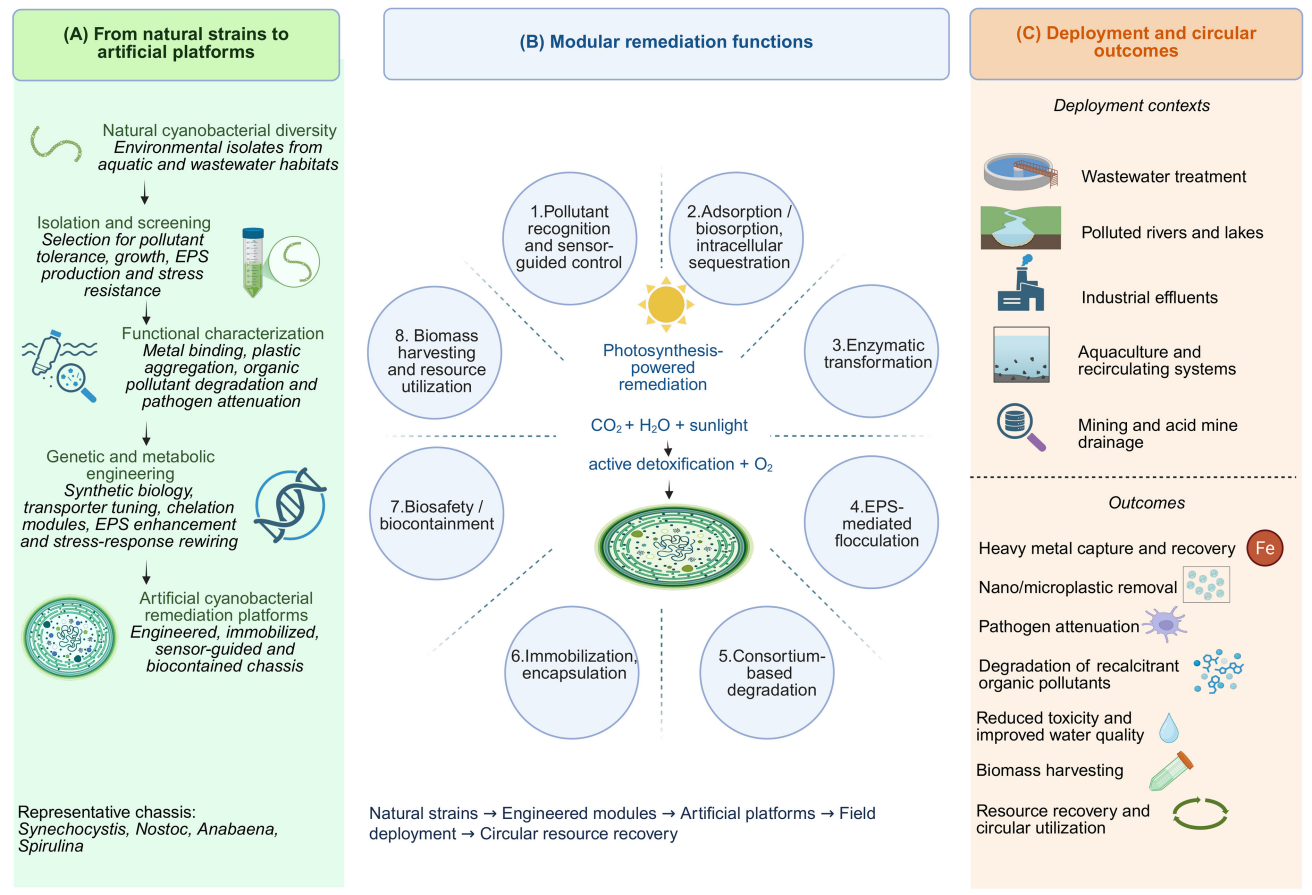
Conceptual roadmap of cyanobacteria-based remediation platforms.

Cyanobacteria are evolutionarily ancient photosynthetic prokaryotic microorganisms capable of converting solar energy into chemical energy through oxygenic photosynthesis while producing oxygen as a byproduct (Nagarajan and Pakrasi, 2016; Nelson and Junge, 2015). These Gram-negative bacteria are the only prokaryotes performing plant-like oxygenic photosynthesis and have existed for more than three billion years (Lau et al., 2015; Nelson and Junge, 2015). **Figure 2** presents the conceptual framework of this review, illustrating the progression from natural cyanobacterial strains to engineered remediation platforms and the major processes involved in pollutant recognition, transformation, immobilization, and resource recovery.

In addition to their fundamental role in global oxygen production and carbon cycling, cyanobacteria possess several physiological characteristics that make them attractive for biotechnological applications. Their relatively simple cellular organization, rapid growth, and ability to utilize carbon dioxide as a primary carbon source allow efficient biomass production under photoautotrophic conditions (Lau et al., 2015). Moreover, these photosynthetic microorganisms require only minimal nutrients for growth and generally possess relatively small and well-characterized genomes, which facilitates their study and manipulation in biotechnology (Zahra et al., 2020).

The broad ecological distribution of cyanobacteria, ranging from aquatic ecosystems to extreme environments, further highlights their remarkable physiological flexibility and adaptive capacity. The success of cyanobacteria in such diverse habitats is associated with a range of physiological and biochemical adaptations, including tolerance to extreme temperatures, desiccation, freezing, salinity stress, and high ultraviolet radiation (Vincent, 2000; Makhalanyane et al., 2015). Additional survival strategies include tolerance to free sulfide and low-oxygen conditions, resistance to intense UV radiation, and, in some cases, the ability to use H_2_S instead of H_2_O as a photoreductant (Pathak et al., 2021). These adaptive traits have enabled cyanobacteria to persist across geological timescales while playing a key role in global biogeochemical cycles and make them attractive candidates for environmental biotechnology applications, including wastewater treatment and the bioremediation of contaminated environments (Stal, 2007; Bhattacharjee et al., 2025).

Cyanobacteria can function as solar-driven platforms for the transformation and detoxification of various anthropogenic pollutants. Through their photosynthetic metabolism, these microorganisms harness light energy to generate ATP and NADPH, which can drive sustainable biotransformation processes without the need for toxic catalysts or high external energy inputs (Cheng et al., 2023). As a result, cyanobacteria are capable of interacting with a wide range of contaminants, including heavy metals, organic pollutants, petroleum-derived compounds, pesticides, and even certain radioactive substances (Kumar and Singh, 2017; Lau et al., 2015). Pollutant removal may occur through several mechanisms, such as bioaccumulation, biosorption, and biodegradation, enabling the conversion of complex toxic compounds into simpler and less harmful forms (Kumar and Singh, 2017). Biosorption occurs at the cell surface through physical adsorption, ion exchange, and complex formation, while bioaccumulation involves intracellular uptake where toxic ions replace essential ions through natural metabolic processes (Cepoi et al., 2016; Al-Amin et al., 2021). Cyanobacteria can contribute to the transformation of complex toxic compounds into simpler and potentially less harmful metabolites, potentially reducing the risk of pollutant accumulation in higher trophic levels (Singh et al., 2023). Their effectiveness stems from metal-binding proteins like metallothioneins and exopolysaccharides that facilitate metal sequestration (Al-Amin et al., 2021).

In addition, their relatively simple growth requirements and genetic tractability make cyanobacteria attractive hosts for environmental biotechnology and metabolic engineering applications (Camsund and Lindblad, 2014; Lau et al., 2015). The combination of photosynthetic energy conversion, metabolic versatility, and pollutant removal capacity positions cyanobacteria as eco-friendly and cost-effective tools for sustainable environmental remediation and pollution control.

Recent advances in molecular biology and metabolic engineering have significantly expanded the potential of cyanobacteria for such applications. Improved genetic engineering tools and synthetic biology approaches, including CRISPR/Cas systems, genome-scale modeling, and high-throughput omics analyses, have enabled more precise modification of cyanobacterial metabolic pathways (Khan et al., 2019; Xia et al., 2019). These developments have led to the creation of sophisticated genetic toolkits comprising optimized promoters, ribosome binding sites, riboswitches, and modular vector systems (Santos-Merino et al., 2019; Xia et al., 2019). As a result, cyanobacteria have been successfully engineered to express heterologous pathways for the production of a wide range of valuable compounds using CO_2_ as a carbon source (Al-Haj et al., 2016; Khan et al., 2019). Nevertheless, further improvements in genetic circuit design and metabolic network integration are still required to make cyanobacterial biotechnology fully competitive with heterotrophic microbial systems (Santos-Merino et al., 2019).

The transition from wild-type cyanobacteria to specialized bioremediation platforms requires a deep restructuring of their internal metabolic architecture. Unlike heterotrophic bacteria, which depend on organic carbon substrates, cyanobacteria utilize solar energy to power complex biochemical transformations, making them ideal candidates for the degradation of energetically “expensive” recalcitrant pollutants. Building upon this photosynthetic foundation, cyanobacteria represent promising platforms for bioremediation, particularly for heavy metal contamination, due to their innate physiological resilience. These microorganisms employ diverse endogenous mechanisms, including biosorption, bioaccumulation, metal transporters, and detoxifying enzymes, to sequester contaminants (Cui et al., 2021; Chakdar et al., 2022).

However, to transcend these natural limitations and achieve industrial-scale efficiency, wild-type strains must be re-engineered into specialized bioremediation chassis. This involves extensive genetic modifications targeting key molecular components such as chaperones, transcriptional regulators, phytochelatins, and metallothioneins (Chakdar et al., 2022). This shift is being catalyzed by recent advances in cyanobacterial omics and synthetic biology, enabling the creation of designer strains tailored for specific environmental applications (Al-Haj et al., 2016). Central to this progress is the vast genetic diversity of cyanobacteria, which provides a “genomic treasure trove” for developing sustainable biotechnology (Selão, 2022).

Synthetic biology tools, including CRISPR-based genome editing, have facilitated the development of engineered cyanobacterial strains with improved pollutant transformation and metal sequestration capabilities under laboratory conditions (Naduthodi et al., 2018; Sengupta et al., 2022).

Building upon the genetic toolkit described above, the true potential of engineered cyanobacteria lies in their ability to act as solar-driven biocatalysts. These photosynthetic prokaryotes can directly convert СО_2_ into valuable compounds and utilize solar energy to support the removal, transformation, and detoxification of recalcitrant pollutants (Knoot et al., 2018; Savakis and Hellingwerf, 2015). By integrating just a few heterologous genes, cyanobacteria can be transformed into “living factories” that produce fuels and commodity chemicals, such as alcohols and terpenoids, directly from atmospheric СО_2_ (Knoot et al., 2018; Savakis and Hellingwerf, 2015). The thermodynamic advantages of this approach are significant: photosynthesis-driven СО_2_ fixation and subsequent metabolic work require minimal external energy compared to conventional physicochemical remediation processes (Kim et al., 2024). However, to fully realize this potential, researchers must address the spectral mismatch the limitation where most cyanobacteria cannot absorb wavelengths >700 nm, effectively bypassing nearly half of the available solar photon flux (Hellingwerf et al., 2021).

Cyanobacteria generate a surplus of reducing equivalents through photosynthesis, which can be strategically redirected to power non-native metabolic pathways. Electron carriers such as ferredoxin and flavodoxin can form productive complexes with heterologous enzymes, most notably hydrogenases and cytochrome P450s (Mellor et al., 2017). Recent engineering breakthroughs have shown that photosynthetic electron transport can be “rewired” to prioritize these artificial sinks. For instance, knocking out NDH-1 subunits involved in cyclic electron flow has been shown to double cytochrome P450 activity while simultaneously increasing cellular ATP levels (Berepiki et al., 2018). Furthermore, implementing heterologous metabolic sinks such as sucrose export or P450 systems can enhance overall photosynthetic capacity by preventing the over-reduction of photosystem I (Santos-Merino et al., 2021). As noted by Hubáček et al. (2024), the cellular NADPH/NADP+ ratio serves as the metabolic conductor, orchestrating the flux of electrons between natural survival pathways and engineered degradation sinks.

This solar-powered redox machinery provides cyanobacteria with a distinct competitive advantage over traditional heterotrophic bacteria in the field of bioremediation. While heterotrophs are limited by the availability of organic carbon substrates, cyanobacteria can degrade pollutants in oligotrophic environments where nutrients are scarce (Kuritz and Wolk, 1995). Their remarkable metabolic flexibility capable of autotrophic, heterotrophic, or mixotrophic growth enables them to persist and function under diverse environmental pressures that might inhibit other microbes (Subashchandrabose et al., 2013). Moreover, these organisms can serve as the cornerstone of synthetic consortia; by providing oxygen and fixed carbon to heterotrophic partners, cyanobacteria facilitate a synergistic environment where complex pollutants are mineralized through collective metabolic labor (Subashchandrabose et al., 2011; Lau et al., 2015). By coupling ecological functions with advances in genetic engineering and metabolic regulation, cyanobacteria extend beyond carbon capture and represent promising biological systems for environmental remediation, although their long-term stability and performance under field conditions require further validation.

## 3 Targeted remediation: cyanobacteria as modular platforms for recalcitrant waste

The metabolic versatility of engineered cyanobacteria allows for the development of specialized “biochemical modules” designed to address specific classes of anthropogenic pollutants. By leveraging solar-driven energy, these organisms move beyond passive filtration toward the active transformation of the most persistent threats to aquatic ecosystems. Cyanobacteria represent a promising approach for active environmental remediation through solar-driven processes that transform persistent aquatic pollutants. These photosynthetic prokaryotes serve as the first biological barrier against toxics entering the food chain and possess inherent capabilities to handle heavy metals and oxidative stress (Cassier-Chauvat and Chauvat, 2014). Recent research demonstrates cyanobacteria’s bidirectional interactions with emerging contaminants, including antibiotics, endocrine disrupting chemicals, persistent organic pollutants, and microplastics, where they can both respond to and actively remediate these pollutants through biosorption, biodegradation, and biotransformation (**Table 1**). Specific studies show cyanobacteria like *Microcystis aeruginosa* can effectively remove high concentrations of antibiotics such as tetracycline (Pan et al., 2021; Yang et al., 2025). Beyond passive filtration, cyanobacteria actively degrade environmental pollutants and remove heavy metals, making them valuable tools for bioremediation and wastewater treatment while utilizing solar energy for carbon-neutral processes (Lau et al., 2015).

**Table 1:** Comparative summary of cyanobacteria-based remediation strategies for different classes of environmental pollutants.

Pollutant category	Representative pollutants	Cyanobacterial species	Strain type	Experimental scale	Pollutant concentration	Removal efficiency/degradation	Mechanism of removal	Key limitations	References
Antibiotics and emerging contaminants	Tetracycline, endocrine-disrupting chemicals	*Microcystis aeruginosa*	WT	Laboratory	High tetracycline concentrations	Effective tetracycline removal	Biosorption, assisted by acoustic cavitation	Limited field validation	Pan et al., 2021; Yang et al., 2025
Microplastics and synthetic polymers	PET, PE microplastics	*Anabaena, Nostoc, Phormidium*	WT	Laboratory	Not specified	70–82% degradation within 8 weeks	Biofilm formation, oxidative polymer degradation	Long treatment period	Sarkhel et al., 2024; Roager and Sonnenschein, 2019
Polyethylene surfaces	PE plastics	*Phormidium lucidum, Oscillatoria subbrevis*	WT	Laboratory	Not specified	Up to 30% weight loss after 42 days	Oxidative biodegradation, carbonyl group formation	Slow degradation rate	Sarmah and Rout, 2018
Engineered plastic degradation	PET polymers	Engineered photosynthetic microorganisms	GE	Laboratory	Not specified	PET degradation	Heterologous enzyme expression	Biosafety and scalability concerns	Dutra Molino et al., 2025
PFAS (fluorinated pollutants)	PFOA, PFOS	*Synechocystis* sp. PCC 6803	WT	Laboratory	Not specified	37% PFOA; 88% PFOS removal	Intracellular sequestration and biotransformation	Incomplete degradation	Marchetto et al., 2021
Chlorinated pesticides	Lindane (γ-HCH)	*Anabaena* sp. PCC 7120, Nostoc ellipsosporum	WT	Laboratory	10 ppm	>98% degradation in 6–10 days	Dechlorination via nitrate reduction system	Mainly laboratory evidence	Kuritz and Wolk, 1995; Chaurasia et al., 2013
Heavy metals	Pb, Cd, Cu, Cr, Hg	EPS-producing cyanobacteria	WT	Laboratory	Variable	Effective metal removal	Biosorption via carboxyl and hydroxyl groups	Metal-specific performance	Pereira et al., 2011; Pembroke et al., 2015
Rare earth elements	Ce, Nd, Tb, La	*Nostoc* sp. 20.02	WT	Laboratory	Not specified	Up to 91.5 mg g^-^¹ adsorption capacity	Ion exchange via EPS functional groups	Limited scale-up studies	Paper et al., 2023
Engineered heavy-metal remediation	Cd²^+^	Engineered *Synechocystis* sp. PCC 6803	GE	Laboratory	Not specified	~21 μg Cd²^+^/OD_750_ removed	Metallothionein and phytochelatin overexpression	Regulatory and biosafety concerns	Sun et al., 2024

The global crisis of plastic pollution, particularly polyethylene terephthalate (PET), requires complementary approaches beyond physical capture, including biological transformation strategies. The global accumulation of PET waste has created a critical ecological crisis, with less than 30% of the 50+ million metric tons produced annually being recycled (Dissanayake and Jayakody, 2021). Traditional physical and chemical recycling methods have significant limitations, prompting research into biological solutions (Liu et al., 2025). Cyanobacteria show potential for microplastic remediation in marine environments through biofilm formation, EPS-mediated aggregation, and polymer transformation. Сyanobacterial species such as *Anabaena* and *Nostoc* have been reported to induce substantial changes in synthetic polymers, including reductions in polymer mass and structural modifications, with reported values of 70–82% under specific laboratory conditions (Sarkhel et al., 2024). However, the extent of complete polymer degradation and mineralization requires further confirmation (Sarkhel et al., 2024). These photosynthetic organisms are considered promising candidates for plastic-associated biotransformation studies due to their ability to capture solar energy and interact with polymer surfaces (Barone et al., 2020). In marine and freshwater environments, cyanobacteria act as pioneer colonizers of microplastics, forming a core component of the “plastisphere” a specialized biofilm community (Zhai et al., 2023). Specific genera, such as *Phormidium*, are consistently identified on marine plastic debris, suggesting a high affinity for polymer surfaces (Roager and Sonnenschein, 2019). This colonization may involve active interactions with polymer surfaces, as laboratory studies have reported that cyanobacterial species within the *Anabaena* and *Nostoc* genera can induce significant polymer modifications, including reductions in polymer mass and structural changes under experimental conditions (Sarkhel et al., 2024).

Because cyanobacteria are obligate photoautotrophs, their role in polymer degradation is primarily restricted to surface-exposed plastic particles in the photic zone (Jacquin et al., 2019). However, their impact on these surfaces is profound. Research involving *Phormidium lucidum* and *Oscillatoria subbrevis* has demonstrated that cyanobacterial interaction with PE surfaces leads to increased hydrophilicity through the formation of carbonyl groupsa key hallmark of oxidative biodegradation (Sarmah and Rout, 2018). Experimental data using standardized PE strips showed a significant rise in the carbonyl index alongside a reduction in polymer crystallinity of up to 62%. Over a six-week incubation, these strains utilized approximately 3–4% of the polymer-bound carbon, resulting in a total weight loss of up to 30% after 42 days (Sarmah and Rout, 2018). These findings suggest that cyanobacteria may contribute to early-stage polymer surface modification and oxidation, potentially increasing polymer accessibility for subsequent microbial processes.

Research demonstrates that photosynthetic microorganisms can be engineered for plastic biodegradation applications. Cyanobacteria, although naturally lacking transporters for certain hydrophilic metabolites, can be metabolically engineered to produce and secrete various compounds through the expression of heterologous transporters together with target enzymes (Niederholtmeyer et al., 2010). This metabolic flexibility, combined with their ability to capture solar energy and CO_2_, makes cyanobacteria promising platforms for sustainable biotechnological applications, including environmental remediation (Barone et al., 2020).

In addition to their potential role in pollutant removal, engineered cyanobacteria can also be used for the biosynthesis of biodegradable polymer precursors such as succinate and lactate, as well as polyhydroxybutyrate (PHB), with genetic modifications significantly enhancing production efficiency (Katayama et al., 2018). Similar synthetic biology strategies have also been applied to other photosynthetic microorganisms. For example, the green microalga *Chlamydomonas reinhardtii* has been engineered to express and secrete the PET-degrading enzyme PHL7, resulting in significant PET transformation was confirmed by mass spectrometric detection of degradation-associated products (Dutra Molino et al., 2025). Together, these studies demonstrate the feasibility of employing engineered photosynthetic microorganisms as sustainable platforms for plastic degradation and environmental bioremediation.

Per- and polyfluoroalkyl substances (PFAS) represent one of the most challenging environmental pollutants due to their exceptional chemical stability. The carbon–fluorine (C–F) bond in PFAS possesses an extremely high bond energy of approximately 116 kCal/mol, which contributes to their persistence and has led to their designation as “forever chemicals” (Noori et al., 2024). This stability, combined with their hydrophobic and lipophobic properties as well as environmental persistence and bioaccumulation potential, makes conventional treatment technologies largely ineffective (Ji et al., 2020).

Recent studies have therefore explored alternative remediation approaches aimed at reducing PFAS concentrations and limiting their environmental mobility. Bioelectrochemical approaches have been explored for PFAS removal, although reported efficiencies and mechanisms vary depending on system design and experimental conditions (Noori et al., 2024). Photosynthetic microorganisms, particularly cyanobacteria, represent promising platforms for such strategies because their photosynthetic electron transport chain provides a continuous and sustainable source of reducing power driven by light energy (Marchetto et al., 2021). Coupling solar-driven electron flow with enzymatic transformation pathways represents a potential strategy for PFAS mitigation, although its effectiveness and mechanisms require further experimental validation (Marchetto et al., 2021; Suzuki et al., 2019).

Among potential candidates, the cyanobacterium *Synechocystis* sp. PCC 6803 demonstrates tolerance to PFAS exposure, surviving at PFOA and PFOS concentrations up to 0.5 and 4 mg L^-^¹, respectively, while showing partial removal rates of 37% for PFOA and 88% for PFOS (Marchetto et al., 2021). The observed removal may involve sorption, accumulation, or transformation processes; however, the underlying mechanisms and potential transformation products remain unclear (Marchetto et al., 2021). Environmental stressors may further influence these interactions; for instance, heatwave conditions combined with PFAS exposure have been reported to stimulate algal growth while simultaneously increasing oxidative stress (Liao et al., 2025). These physiological and ecological responses highlight the value of microalgae as bioindicators of PFAS contamination and provide insights into their interactions with PFAS in aquatic ecosystems, although their practical application in remediation requires further investigation (Li et al., 2025).

The evidence supporting plastic and PFAS transformation varies depending on the analytical approaches applied. For PFAS, studies such as Marchetto et al. (2021) primarily reported reductions in aqueous PFAS concentrations and cellular responses. Similarly, plastic-associated studies have mainly evaluated polymer changes through parameters such as mass reduction, surface modification, or chemical alterations rather than complete mineralization confirmed by CO_2_ evolution or carbon balance analysis. Therefore, reported improvements in pollutant removal should be interpreted in relation to the underlying mechanisms, including adsorption, sequestration, surface modification, and partial transformation.

Similarly, the potential of cyanobacteria for pollutant remediation has also been demonstrated for other persistent halogenated compounds, including chlorinated pesticides. Photoautotrophic microorganisms, particularly filamentous cyanobacteria, demonstrate natural abilities to degrade chlorinated pollutants including highly chlorinated pesticides like lindane. Kuritz and Wolk (1995) showed that two filamentous cyanobacteria species possess inherent capacity to degrade lindane (gamma-hexachlorocyclohexane) and can be genetically engineered to enhance this ability and degrade other chlorinated compounds like 4-chlorobenzoate. Kuritz et al. (1997) identified fifteen cyanobacterial strains capable of lindane degradation, with filamentous nitrogen-fixing strains *Anabaena* sp. PCC 7120 and *Nostoc ellipsosporum* dechlorinating lindane to pentachlorocyclohexene and subsequently to trichlorobenzenes. This dechlorination process required nitrate presence and was inhibited by ammonium and darkness, suggesting involvement of the nitrate-reduction system. Chaurasia et al. (2013) demonstrated that engineered *Anabaena* could degrade >98% of 10 ppm lindane within 6–10 days.

In addition to organic pollutants, cyanobacteria also exhibit significant potential for the remediation of inorganic contaminants such as heavy metals. Heavy metals including lead, cadmium, mercury, and chromium represent priority pollutants in surface and groundwater due to their high toxicity even at trace concentrations (Atieh et al., 2017). Unlike organic contaminants, heavy metals are non-biodegradable and tend to accumulate in living organisms, making them persistent environmental threats (Atieh et al., 2017; Azimi et al., 2017). Industrial activities such as mining, electroplating, and battery manufacturing are major sources of these pollutants in aquatic ecosystems (Jadaa and Mohammed, 2023).

Cyanobacteria can contribute to heavy metal removal through biosorption mechanisms mediated by EPS. These polysaccharidic matrices contain negatively charged functional groups that facilitate metal binding through passive adsorption and complexation (Pereira et al., 2011). EPS producing cyanobacteria have been shown to effectively remove various metals including Pb, Cu, Cd, Cr, and Hg from aqueous environments (Pembroke et al., 2015). The biosorption efficiency varies depending on the metal type; for instance, Pb²^+^ can be preferentially adsorbed over Cu²^+^ in multi-metal systems (Pereira et al., 2011). *Spirulina platensis* biomass demonstrates significant potential as a biosorbent for metal immobilization and removal from aqueous systems, including cobalt and uranium. Zinicovscaia et al. (2016) showed that *S. platensis* effectively accumulates uranium from both single and multi-component systems, with biosorption rates varying based on system composition. The biomass functions as a low-cost sorbent for industrial wastewater treatment. Vannela and Verma (2008) demonstrated that *S. platensis* achieves high cobalt biosorption capacity (181.0 mg Co²^+^/g with living cells), with optimal performance at pH 6.0 and rapid initial uptake phases contributing 63-77% of total biosorption within 1–2 minutes. The biomass has demonstrated potential for reuse in repeated biosorption cycles under optimized experimental conditions. Gadd (2009) highlighted biosorption as a promising approach for the removal of dissolved metals and radionuclides from contaminated systems. Huertas et al. (2014) emphasize that understanding cyanobacterial metal homeostasis mechanisms, including cobalt metabolism, is crucial for evaluating their bioremediation applications.

Metal tolerance is associated with membrane transporters, efflux systems, and intracellular metal-binding proteins (Hossain and Okino, 2024). Metallothioneins bind metals via cysteine thiol groups and are induced by elevated metal concentrations (Al-Amin et al., 2021). Cytoplasmic sequestration is further supported by these proteins and metal–ligand complex formation. Polyphosphate (Poly-P) bodies may function as intracellular metal-binding sites and contribute to metal homeostasis under stress conditions (Sanz-Luque et al., 2020). Metal ions may also associate with thylakoid systems, linking metal handling with bioenergetic processes (Kalita et al., 2026).

Genomic and proteomic advances have enabled identification of regulatory networks governing metal stress. Overexpression of antioxidant enzymes such as superoxide dismutases (SodA, SodC) and catalase enhances ROS detoxification and improves tolerance (Yang et al., 2026). Exogenous genes, including metallothioneins, the manganese transporter (MntH), and SOD genes, improve resistance to cadmium and mixed-metal stress. Engineered *Synechocystis* sp. PCC 6803 strains incorporating phytochelatin and metallothionein pathways show enhance metal tolerance and increase cadmium association or uptake capacity (Sun et al., 2024). However, synergistic multi-gene effects remain insufficiently characterized.

Cyanobacteria have demonstrated the capacity to reduce dissolved cadmium concentrations under laboratory conditions (Ahad et al., 2017). Hossain and Okino (2024) reported 78% removal of Cd²^+^ at an initial concentration of 1 mg/L using *Anabaena variabilis*, highlighting the efficiency of biosorption at low concentrations (Hossain and Okino, 2024). Similarly, others observed a 57% removal by *Synechocystis* sp. under identical conditions, suggesting species-specific variability in uptake efficiency (Chakdar et al., 2022).

Beyond removal efficiency, cadmium exposure has been shown to significantly impact cyanobacterial physiology. Abd El-Hameed et al. (2021) reported that Cd²^+^ exposure caused marked reductions in chlorophyll and carotenoid content in *T. variabilis* and *N. muscorum* after 14 days, indicating disruption of the photosynthetic machinery. At the cellular level, Ruan et al. (2022) demonstrated that excessive Cd²^+^ influx in *Synechocystis* sp. PCC 6803 leads to elevated ROS production, which triggers increased superoxide dismutase (SOD) activity but ultimately results in lipid peroxidation and structural damage to thylakoid membranes.

EPS composition plays a critical role in cadmium sequestration. The presence of uronic acids such as glucuronic and galacturonic acid enhances binding affinity and improves removal efficiency (Singh et al., 2023). Additional studies have shown that cadmium uptake can be enhanced under optimized pH (typically mildly acidic to neutral), increased biomass dosage, and extended contact times (Gupta and Rastogi, 2008; Wang and Chen, 2009).

Cyanobacteria exhibit both biosorption and enzymatic detoxification capabilities for mercury removal. *Spirulina platensis* has demonstrated removal efficiencies of up to ~98%, characterized by rapid initial adsorption followed by slower intracellular uptake phases (Yadav et al., 2022; Kalita et al., 2025).

Franco et al. (2018) reported that *Nostoc paludosum* achieved 73-96% removal of inorganic mercury and 73-95% removal of organic mercury species from wastewater, underscoring the versatility of cyanobacterial systems. The presence of thiol-rich binding sites in metallothioneins contributes significantly to mercury sequestration (Franco et al., 2018).

Importantly, organomercury lyase systems enable the breakdown of methylmercury into less toxic forms. These enzymatic pathways, coupled with mer-operon regulatory systems, provide a genetic basis for engineering enhanced mercury detoxification systems.

Cyanobacteria have shown strong potential for the removal of uranium and other radionuclides, which are major contaminants associated with nuclear energy, mining, and industrial processes. Uranium removal is primarily driven by phosphate-mediated binding, surface adsorption, and intracellular sequestration mechanisms (Mohammad et al., 2025).

Ismaiel et al. (2022) reported 60% uranium biosorption by *Nostoc* sp. at an initial concentration of 300 mg/L within 60 minutes at pH 4.5 using a biomass dosage of 4.2 g/L. The authors also noted that interactions with other metals (e.g., Cd, Co, Ni) may influence uptake efficiency. Smječanin et al. (2023) demonstrated strong uranium affinity in *Anagnostidinema amphibium* at 100 mg/L across alkaline conditions (pH 9–11), suggesting pH-dependent binding mechanisms.

Immobilized systems further enhance uranium removal capacity. Mohammad et al. (2025) reported that immobilized *Nostoc* sp. achieved biosorption capacities of approximately 75 mg/g, indicating strong potential for scale-up applications. Polyphosphate accumulation has been identified as a key mechanism for radionuclide sequestration, with phosphate groups facilitating strong coordination and immobilization of uranium ions through uranyl-phosphate complex formation (Acharya et al., 2009; Acharya and Apte, 2013; Chandwadkar and Acharya, 2023).

While many studies focus on single-metal systems, real wastewater streams typically contain complex mixtures of metals. Cyanobacteria have demonstrated the ability to simultaneously remove multiple metal ions, although competitive binding effects may influence efficiency. Studies have shown that co-presence of metals can either enhance or inhibit biosorption depending on ionic interactions, binding site availability, and environmental conditions (Volesky, 2007). In addition, factors such as pH, ionic strength, temperature, and presence of organic matter significantly impact removal efficiency. Continuous-flow systems and immobilized-biomass approaches have been explored to improve stability and scalability under realistic conditions. These findings highlight the importance of translating laboratory-scale performance into practical, field-relevant systems.

The metal-binding mechanism is primarily associated with carboxyl groups located on cyanobacterial cell surfaces and within EPS matrices, where deprotonation reactions lead to increased COO^-^ formation and enhanced metal complexation (Yee et al., 2004). In addition, metal exposure may stimulate EPS production and modify its biochemical composition. For example, *Synechocystis* sp. BASO671 exposed to chromium and cadmium exhibited increased uronic acid content in EPS, which correlated with improved metal removal capacity (Ozturk et al., 2014). These findings highlight the potential of EPS-producing cyanobacteria as effective biological agents for heavy-metal remediation in contaminated environments (De Philippis et al., 2011).

Beyond the sequestration of dissolved ions, the versatile physicochemical properties of these matrices allow cyanobacteria to address larger particulate threats, such as synthetic polymers. Specifically, cyanobacterial EPS show significant promise as bio-based flocculants for removing polystyrene microplastics from water. Research demonstrates that EPS from *Cyanothece* sp. exhibits high bioflocculant activity at low concentrations, effectively aggregating microalgae, EPS, and both nano- and microplastics into removable clusters (Cunha et al., 2020). Similarly, soluble EPS from *Cyanocohniella rudolphia* successfully removes polystyrene microplastics, with production enhanced by microplastic exposure, enabling circular reuse strategies that reduce reliance on synthetic coagulants (Rodrigues et al., 2025).

These cyanobacterial EPS, composed primarily of heteropolysaccharides, represent sustainable alternatives to synthetic polymers and can be strategically designed through genetic engineering or chemical modification (Pereira et al., 2019). The flocculation mechanisms involve polysaccharides, proteins, lipids, and DNA constituents, making microalgal EPS valuable bioproducts for both biomass harvesting and wastewater treatment applications (Babiak and Krzemińska, 2021).

Cyanobacteria possess sophisticated metal homeostasis mechanisms that enable them to manage both essential and toxic metals in their cellular environment. These organisms require several metals for physiological processes, including copper for plastocyanin, zinc for carbonic anhydrase, and cobalt for cobalamin synthesis (Cavet et al., 2003). To cope with metal stress, cyanobacteria employ multiple strategies such as biosorption, bioaccumulation, and the activation of specialized transport systems and metallochaperone proteins that regulate intracellular metal concentrations and prevent toxicity (Finney and O’Halloran, 2003; Huertas et al., 2014). Important detoxification components include metal-binding peptides such as metallothioneins (MTs) and phytochelatins (PCs), which chelate excess metal ions and contribute to cellular metal homeostasis (Balzano et al., 2020; Chakdar et al., 2022).

Building on these natural mechanisms, genetic engineering approaches have been developed to enhance cyanobacterial heavy metal bioremediation. Overexpression of MT and PC genes has been proposed as a key strategy for improving metal-binding capacity (Chakdar et al., 2022). For example, Sun et al. (2024) engineered *Synechocystis* sp. PCC 6803 with MT and PC genes, generating strain PM/6803 that exhibited increased tolerance to multiple heavy metals and achieved removal rates of approximately 21 μg Cd²^+^/OD_750_. Encapsulation of this engineered strain in alginate hydrogels further enabled effective cadmium removal in zebrafish and mice models, demonstrating potential for practical applications.

In addition to engineered systems, several cyanobacterial strains exhibit naturally high metal adsorption capacities. Screening of twelve strains revealed adsorption capacities of 84.2–91.5 mg g^-^¹ for rare earth elements such as cerium, neodymium, terbium, and lanthanum, with *Nostoc* sp. 20.02 showing the highest performance (Paper et al., 2023). This biosorption process occurs primarily through ion-exchange interactions involving functional groups such as hydroxyl and carboxyl groups, typically under optimal pH conditions of 5–6 (Paper et al., 2023).

Beyond environmental remediation, metal-enriched cyanobacterial biomass can be valorized into value-added products such as metal nanoparticles, contributing to the emerging field of phyconanotechnology and supporting circular economy strategies (Ciani and Adessi, 2023). This approach offers environmentally friendly and cost-effective alternatives to conventional remediation technologies while enabling recovery of critical materials such as gold and lanthanum from contaminated environments (Birungi et al., 2020; Oziegbe et al., 2025).

Despite the promising potential of cyanobacteria for heavy metal removal, the effectiveness and practical applicability of these systems vary depending on the pollutant type, cyanobacterial species, experimental design, and degree of technological development. Most available studies have been conducted under controlled laboratory conditions, where biosorption, bioaccumulation, and metal-binding mechanisms can be efficiently evaluated. However, the transition from laboratory-scale experiments to pilot-scale and field applications remains limited and requires further validation. A comparative overview of cyanobacteria-based remediation approaches, including experimental systems, evidence levels, and development stages, is summarized in **Table 2**.

**Table 2:** Current status of cyanobacterial applications in pollutant removal: experimental systems, evidence level, and technological readiness.

Pollutant	Experimental system	Evidence level	Development stage	Key references
Heavy metals	Laboratory cultures, pilot-scale systems	High	Most mature application area	Pereira et al., 2011; Pembroke et al., 2015; Hossain and Okino, 2024
Pesticides	Laboratory cultures, microbial consortia	Moderate	Requires further scaling and validation	Kuritz and Wolk, 1995; Chaurasia et al., 2013
Microplastics	Mainly laboratory-scale studies	Low–moderate	Early-stage research	Sarmah and Rout, 2018; Sarkhel et al., 2024
PFAS (fluorinated contaminants)	Limited number of laboratory-scale studies	Low	Conceptual stage	Marchetto et al., 2021; Noori et al., 2024

Assessment was based on available evidence, experimental scale, reproducibility of results, and degree of validation beyond laboratory conditions.

However, cyanobacterial-based approaches face several limitations that may affect their practical application in metal remediation. Heavy metals can adversely affect cyanobacterial growth and development by disrupting key physiological processes, photosynthetic activity, and biomass accumulation (Oziegbe et al., 2025). In addition, the efficiency of cyanobacterial metal removal may vary depending on environmental conditions, including light intensity, UV exposure, nutrient availability, temperature, and salinity (Paper et al., 2023).

Although cyanobacteria possess molecular defense mechanisms such as metal-binding proteins, transport systems, and antioxidant responses, important knowledge gaps remain regarding long-term tolerance, metal recovery, and performance under environmentally relevant conditions, limiting their broader application in remediation programs (Oziegbe et al., 2025). Therefore, despite promising laboratory-scale results, further validation using pilot-scale systems and realistic environmental conditions is required to evaluate the scalability and practical feasibility of cyanobacteria-based metal remediation technologies.

## 4 Engineering strategies to enhance cyanobacteria-based bioremediation

Synthetic biology provides powerful toolkits for engineering cyanobacteria into efficient biotechnological platforms that can overcome natural limitations. These photoautotrophic microorganisms are ideal chassis for the direct conversion of CO_2_ and solar energy into valuable products due to their photosynthetic capabilities (Xia et al., 2019; Wang et al., 2020). Recent advances include the development of genetic toolkits encompassing heterologous gene expression systems, genome editing strategies, and programmable regulatory modules that enable the implementation of non-natural functionalities (Xia et al., 2019; Santos-Merino et al., 2019). These tools facilitate the engineering of cyanobacteria for the sustainable production of biofuels, biochemicals, and living biomaterials, including bioconcrete, biocomposites, and biophotovoltaics (Goodchild-Michelman et al., 2023). The synthetic biology approach incorporates advances in genetic promoters, ribosome binding sites (RBS), riboswitches, and modular vector systems that enhance both controllability and robustness (Santos-Merino et al., 2019).

Despite these technological advancements, a fundamental bottleneck remains: the expression of heterologous enzymes in bioremediation applications creates a significant metabolic burden on host microorganisms, often limiting their effectiveness in the field. This metabolic load occurs when foreign protein expression diverts cellular resources such as amino acids, energy, and precursors away from normal host metabolism, dramatically altering the biochemistry and physiology of the organism and reducing target protein production (Glick, 1995). In bioremediation contexts, cyanobacteria must maintain complex metabolic networks to process pollutants while simultaneously managing primary production. This requires precise fine-tuning of central pathways, including the tricarboxylic acid (TCA) cycle, glycolysis, and the pentose phosphate pathway, to generate essential cofactors like NADPH and ATP (Tharmalingam et al., 2017). While genetic engineering has successfully enhanced the expression of enzymes for metal transformation and xenobiotic degradation (Jan et al., 2014), and cell surface display technologies have emerged for heavy metal detoxification (Saleem et al., 2008), the core challenge persists: heterologous expression imposes metabolic constraints that must be strategically managed.

**Figure 3:**
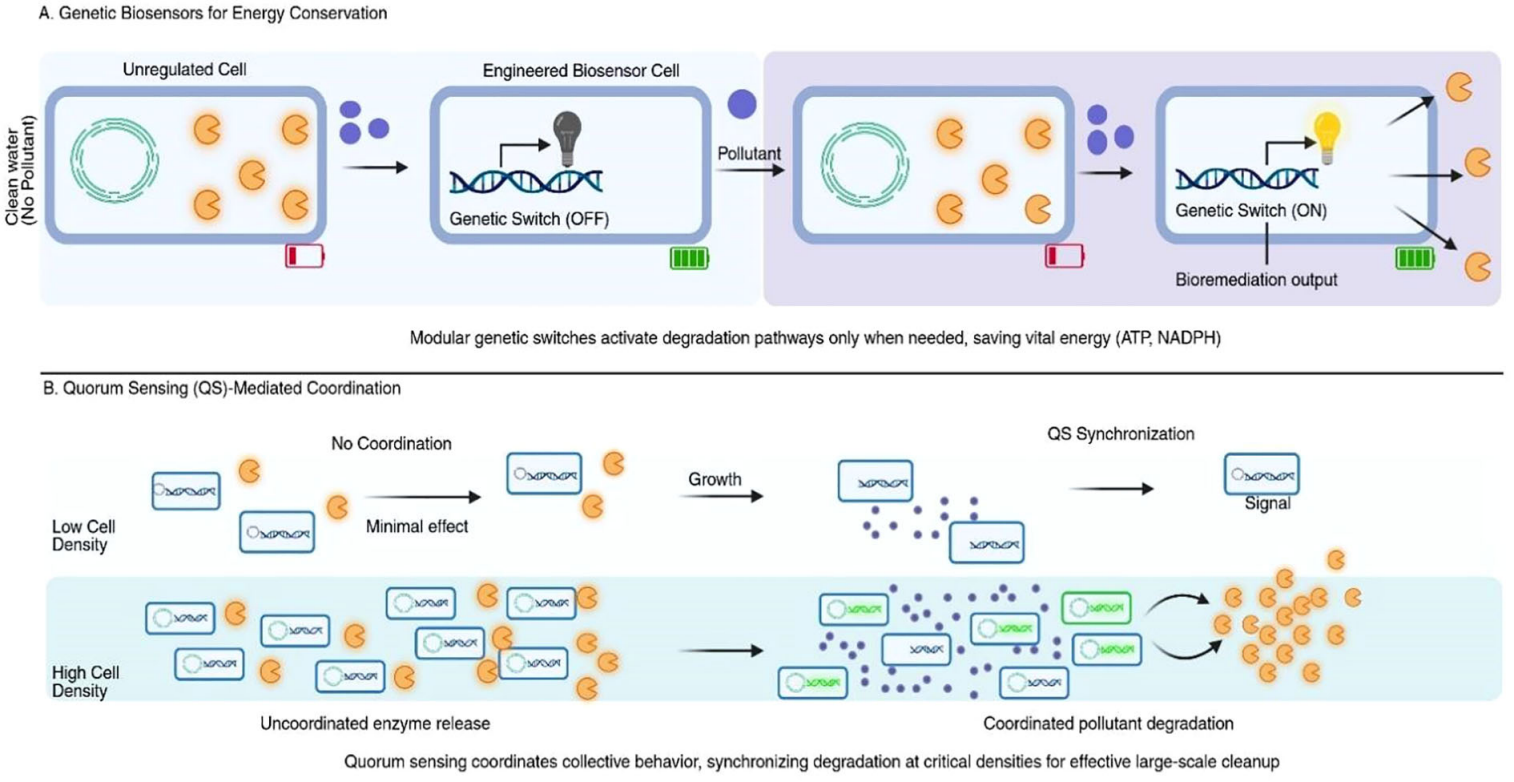
Smart control strategies for optimized bioremediation. This simplified schematic illustrates two key approaches. **(A)** Modular genetic switches (biosensors) function as dynamic control systems, ensuring that degradative enzymes are synthesized *only* in response to a pollutant trigger, thereby conserving cellular energy (ATP, NADPH). **(B)** Quorum sensing (QS) enables collective behavior, synchronizing the activation of degradation across the entire population at critical cell densities for enhanced large-scale efficiency.

To mitigate this metabolic strain, researchers have moved toward “conditional” expression systems using whole-cell microbial biosensors. These biosensors act as cost-effective tools for detecting environmental pollutants through engineered genetic circuits, allowing degradative pathways to be activated only in the presence of the target contaminant. These systems utilize transcription factors from bacterial resistance mechanisms to sense heavy metals (Hg²^+^, Zn²^+^, Pb²^+^, Cd²^+^, etc.) and organic compounds such as alcohols, alkanes, and phenols (Liu et al., 2022). Current designs incorporate complex components including toggle switches, logic gates, and amplification modules to enhance sensitivity and specificity (Mathur et al., 2023). Optimization strategies focus on selectivity through protein engineering and circuit-based amplification using feedback loops (Liu et al., 2022; Bernard and Wang, 2017). Novel designs, such as recombinase-based circuits (e.g., Bxb1 recombinase), provide tighter control and reduced background signals, as demonstrated in cadmium detection systems (Akboğa et al., 2022). The operational logic of these genetic switches in mitigating metabolic strain by activating degradation pathways only upon pollutant detection is illustrated in **Figure 3A**.

Within the specific context of aquatic monitoring, cyanobacterial biosensors offer a unique advantage by integrating directly with the photosynthetic machinery. For instance, Shao et al. (2002) developed a bioluminescent biosensor by marking *Synechocystis* sp. PCC 6803 with the firefly luciferase gene, enabling the detection of various herbicides through bioluminescence. Earlier work by Rawson et al. (1987, 1989) demonstrated that mediated amperometric biosensors using *Synechococcus* could detect herbicides at concentrations below 200 ppb with rapid response times by monitoring photosynthetic electron transfer. These amperometric systems, often utilizing alginate immobilization, achieve high sensitivity (20 μg/L) and operational lifespans of up to 7 days. The reliability of these photosynthetic platforms has been confirmed through multi-laboratory evaluations, validating their potential for real-world water pollution monitoring (van Hoof et al., 1992).

Beyond individual cell sensing, the coordination of the entire population through Quorum Sensing (QS) provides the final layer of metabolic optimization. QS systems enable population density-dependent coordination of gene expression, supporting the conservation of metabolic energy until critical cell densities are reached. Kokarakis et al. (2025) demonstrated that engineered QS circuits in *Synechococcus elongatus* PCC 7942 can produce and detect acyl-homoserine lactones (AHLs) to activate gene expression in a dose-dependent, responsive manner. This autoinduction is essential for large-scale applications, where synchronized biological activity is required to process high volumes of waste. Furthermore, QS allows for community-level metabolic reprogramming, shifting from individual energy generation to community-preserving modes that offset the accumulation of toxic metabolites at high densities (Hawver et al., 2016). This population-level coordination provides the necessary foundation for developing scalable, autonomous cyanobacterial biotechnology platforms for global remediation efforts (Kokarakis et al., 2025). The transition from isolated cell activity to synchronized community-wide degradation through these quorum sensing mechanisms is schematically represented in **Figure 3B**.

While individual engineered strains are powerful, complex pollutants such as petroleum hydrocarbons often require multi-step enzymatic attacks that exceed the metabolic capacity of a single species. To overcome these limitations, the future of bioremediation lies in synthetic microbial consortia (SMC). Unlike natural microbial communities, which may not be optimally structured for anthropogenic waste degradation, synthetic consortia can be precisely engineered to optimize metabolic labor and reconfigure pathways for specific environmental outcomes (Chen et al., 2025; Li et al., 2021). By mastering these inter-species interactions, bioengineering transforms cyanobacteria from isolated cells into the “solar-powered life support” of self-sustaining remediation ecosystems.

In these ecological frameworks, cyanobacteria act as the primary engines by providing essential resources to heterotrophic partners. Through oxygenic photosynthesis, they supply the oxygen required by heterotrophs for the oxidative degradation of organic pollutants, while receiving CO_2_ and essential growth factors in return (Subashchandrabose et al., 2011). Furthermore, cyanobacteria can be engineered as “provider” strains to export fixed carbon. For instance, engineered *Synechococcus elongatus* PCC 7942 can export up to 85% of its photosynthetically-fixed carbon as sucrose, enabling the growth of diverse heterotrophs such as *Bacillus subtilis*, *Escherichia coli*, and *Saccharomyces cerevisiae* in minimal media without external carbon sources (Weiss et al., 2017; Zuñiga et al., 2020).

The diversity and practical implementation of these collaborative systems are summarized in **Table 3**, highlighting how metabolic synergy creates a robust platform for treating recalcitrant waste.

**Table 3:** Applications and mechanisms of cyanobacterial–bacterial consortia in bioremediation.

Cyanobacterial partner	Bacterial/microbial partner	Target pollutant	Consortium type	Experimental scale	Performance outcome	Mechanism of interaction	Key limitations	Reference
Synechococcus elongatus	Pseudomonas putida	2,4-Dinitrotoluene (DNT) degradation	Engineered	Laboratory	Effective DNT degradation	Cyanobacteria provide fixed carbon and oxygen supporting heterotrophic degradation	Limited field validation	Fedeson et al., 2020
Mixed cyanobacteria consortia	Native wastewater bacteria	Sulfamethoxazole-contaminated wastewater	Natural	Laboratory/Pilot	Simultaneous antibiotic degradation and nutrient removal	Cyanobacteria stimulate bacterial enzymatic activity and nutrient removal	Variable wastewater composition	Fang et al., 2024
Cyanobacteria/microalgae consortia	Mixed heterotrophic bacteria	Petroleum hydrocarbons and xenobiotics	Natural	Laboratory	Enhanced hydrocarbon degradation	Photosynthetic oxygen production and metabolite exchange	Community instability under environmental stress	Subashchandrabose et al., 2011
Cyanobacterial consortia	Heterotrophic wastewater bacteria	Wastewater treatment and CO_2_ capture	Natural	Pilot	Simultaneous pollutant removal and carbon capture	Cyanobacteria support microbial degradation while fixing CO_2_	Environmental variability	Lazaratou et al., 2024
Cyanobacteria–bacterial consortia	Mixed microbial communities	Nutrient removal and wastewater treatment	Natural	Pilot	Improved N and P removal and biomass production	Metabolic cooperation between consortium members	Operational complexity	Li et al., 2024
Microalgae/cyanobacteria consortia	Environmental microbial communities	Wastewater remediation and bioenergy production	Natural	Pilot	Enhanced biomass production and pollutant removal	Cooperative metabolism supports biofuel generation	Economic scalability challenges	Mehra et al., 2025

A notable success is the co-culture of *S. elongatus* and engineered *Pseudomonas putida*, which achieved the phototrophic degradation of 2,4-dinitrotoluene while simultaneously producing bioplastic (Fedeson et al., 2020). In hydrocarbon-degrading systems, species like *Microcoleus chthonoplastes* provide both oxygen and organic matter within their polysaccharidic sheaths to support oil-degrading bacteria (Sánchez et al., 2005). Moreover, mixotrophic cyanobacteria can act as distinctive agents by simultaneously achieving carbon sequestration and organic pollutant degradation (Subashchandrabose et al., 2013), as demonstrated by the removal of ammoniacal nitrogen, phenol, and nitrate from coke-oven wastewater under mixed-culture conditions (Rai et al., 2020).

Ultimately, the modularity of these consortia allows for a “plug-and-play” integration of specialized bacteria, offering significant advantages over monocultures. Metabolic modeling reveals that these synthetic communities effectively bypass species-specific bottlenecks and can compensate for lethal genetic traits, achieving up to 27% recovery from lethal knockouts (Zuñiga et al., 2020). Despite challenges from heavy metal toxicity and reactive oxygen species (ROS), the division of labor within these consortia provides the resilience needed to endure environmental fluctuations, marking a definitive shift toward more robust and sustainable biodegradation platforms (Adamu et al., 2023; Brenner et al., 2008).

While synthetic consortia have shown potential for enhancing metabolic cooperation, their practical deployment in open environments remains limited by challenges related to physical stability, washout, and long-term system performance. Cell immobilization and surface engineering address these critical challenges by providing a structural framework that mimics natural cellular organization while enhancing functional durability (Dulieu et al., 1999). By shielding engineered cells from shear stress and predation, immobilization enables microorganisms to demonstrate superior resistance to toxic chemicals, pH fluctuations, and temperature changes compared to suspended systems (Martins et al., 2013; Partovinia and Rasekh, 2018).

Surface engineering through biomimetic coatings further complements this protection, dramatically increasing cell viability in hostile environments by providing mechanical stability and masking cells from aggressive external agents (Drachuk et al., 2013). Advanced strategies now involve genetically engineering bacterial biofilms to anchor functional nano-objects. This creates scalable and reusable catalytic systems that effectively segregate high-energy nanomaterials from cellular components through extracellular matrix anchoring, thus maximizing efficiency while minimizing nanotoxicity (Wang et al., 2019).

The physical confinement provided by immobilization also directly supports the genetic control mechanisms discussed previously. By maintaining high cell densities within the matrix, these systems facilitate the robust quorum sensing (QS) signaling required to synchronize the metabolism of toxic compounds (Mangwani et al., 2016). This spatial organization significantly boosts performance; studies have shown that immobilized microorganisms can achieve removal efficiencies over 21% higher than free consortia, with certain applications reaching complete pollutant removal (Najim et al., 2024). The practical success of these systems is heavily dependent on the choice of immobilization matrix, which determines both the longevity and the chemical efficiency of the process. For instance, magnesium phosphate-based cements have been successfully employed as a stable matrix, maintaining cyanobacterial viability and metabolic activity for at least four weeks (Vorndran and Lindberg, 2016).

Furthermore, the choice of material directly impacts the removal capacity for specific toxins. Sodium alginate immobilization has demonstrated exceptional efficiency in heavy metal sequestration; studies using *Anabaena variabilis* and *Tolypothrix ceytonica* achieved removal rates reaching up to up to 94.45% for Fe²^+^, 94.22% for Pb²^+^, and 93.33% for Cu²^+^ (El Bestawy, 2019). Similarly, silica hybrid matrices have proven effective for targeted remediation, with immobilized *Synechocystis salina* achieving significant adsorption capacities, specifically 7.6 mg/g for copper and 9.0 mg/g for cadmium (Toncheva-Panova et al., 2010). Beyond pollutant removal, these immobilization strategies facilitate a broader industrial objective: the transition from waste treatment to resource recovery. By enabling the easy collection of metal-saturated biomass, these processes support metal valorization and circular economy principles, providing the economic feasibility necessary for large-scale wastewater treatment applications (Ciani and Adessi, 2023).

## 5 Challenges and limitations for field-scale implementation of cyanobacterial remediation systems

Despite their promising performance under controlled laboratory conditions, the field-scale implementation of cyanobacterial remediation systems faces several significant challenges. The efficiency and long-term stability of these systems are strongly influenced by environmental conditions, which can differ substantially from those used in laboratory experiments (Grettenberger et al., 2024). In addition, cyanobacteria generally exhibit slower growth rates and lower biomass productivity than many heterotrophic microorganisms, which may limit remediation rates and prolong treatment times under large-scale applications (Srivastava et al., 2021). Light intensity significantly influences the physiological state of cyanobacteria, photosynthetic processes, the generation of reactive oxygen species, and the efficiency of pollutant removal. Variations in light conditions can both stimulate cyanobacterial growth and metabolic activity and induce oxidative stress, thereby reducing the effectiveness of bioremediation processes (Grossman et al., 2001; Rahman et al., 2023). Temperature is another key environmental factor affecting cyanobacterial physiology, growth, and metabolic activity, thereby influencing their performance in biotechnological and bioremediation applications. Elevated temperatures can induce physiological stress, resulting in reduced growth, increased oxidative damage, and decreased nitrogenase activity despite enhanced antioxidant responses (Reddy et al., 2019). Conversely, thermotolerant cyanobacterial strains isolated from hot springs exhibit enhanced stress tolerance, higher growth rates, and greater antioxidant capacity under elevated temperatures compared with mesophilic strains (Amin et al., 2024). These findings indicate that temperature is a critical factor influencing the efficiency, stability, and scalability of cyanobacteria-based remediation systems under field conditions. pH requirements vary across growth phases, and cyanobacteria can modify water pH to favor their own growth (Cuichao et al., 2013). Nutrient availability, particularly nitrogen-to-phosphorus ratios, significantly impacts growth rates and competitive advantage (Cuichao et al., 2013). Additional limiting factors include UV exposure, salinity, and CO_2_ concentrations, with synergistic interactions between these parameters affecting both growth and secondary metabolite production (Grettenberger et al., 2024). In real wastewater and natural aquatic systems, pollutant removal may be further complicated by the presence of competing ions, dissolved organic matter, suspended solids, and mixtures of contaminants that can alter pollutant bioavailability and interfere with removal mechanisms (Torekhanova et al., 2023). Biological interactions, including microbial competition, grazing, and contamination by indigenous microorganisms, may also affect the stability and persistence of cyanobacterial populations.

In addition to environmental constraints, several engineering and operational considerations must be addressed. These include optimization of large-scale cultivation systems, contamination control, hydraulic retention time, biomass harvesting, downstream biomass processing, and the safe disposal or recovery of accumulated pollutants. For salt-affected soil remediation, key requirements include the selection of suitable cyanobacterial strains and the development of cost-effective cultivation strategies (Li et al., 2019). In aquatic systems, treatment performance may be strongly influenced by the limnological and hydrobiological characteristics of water bodies, including hydrology, nutrient dynamics, and continuous external nutrient inputs (Kibuye et al., 2021). Cyanobacteria can effectively remove persistent organic pollutants including phenols, pesticides, and polychlorinated biphenyls, with optimal growth requiring specific nutrient compositions in cultivation media (Cepoi et al., 2016). Chemical control strategies for managing cyanobacterial populations are influenced by water quality conditions and source water characteristics such as surface area, depth, and residence time (Kibuye et al., 2021).

Research demonstrates that biodegradation and pollutant removal processes are significantly scale-dependent, with field-scale effectiveness often reduced compared to laboratory studies. Scale-dependent variables including mass transport limitations, spatial heterogeneities, and competing microorganisms can inhibit field-scale bioremediation effectiveness (Sturman et al., 1995). Laboratory investigations typically operate at small scales for convenience and cost, but scaling up introduces additional mass transport mechanisms, multiple phases, spatial geologic heterogeneities, and subsurface environmental factors that may inhibit bacterial growth (Li et al., 2019). Experimental evidence shows that biodegradation rates slow as experimental scale increases, with high variability resulting from small-scale heterogeneities, making small-scale experimental rates unrepresentative of field-scale conditions (Davis et al., 2003). Limited pollutant accessibility due to unequal spatial distribution of microorganisms and pollutants, combined with substrate diffusion retardation by soil matrix, contributes to slower biodegradation rates than expected from laboratory trials (Davis et al., 2003). Therefore, additional pilot-scale and field-scale investigations are required to evaluate the long-term stability, scalability, and practical feasibility of cyanobacterial remediation technologies. Furthermore, integrated studies that combine environmental, biological, and engineering factors will be essential for the successful transition of cyanobacterial remediation systems from laboratory research to real-world applications.

## 6 Perspectives, ethics, and the future of circular bioeconomy

The transition from laboratory-scale engineered cyanobacteria to real-world bioremediation faces significant regulatory and practical challenges. Current deployment remains stalled due to outdated containment-centric frameworks; as noted by de Lorenzo (2026), a shift from control-based to stewardship-based approaches is required. While genetic modifications have successfully improved cadmium removal and tolerance (Sun et al., 2024; Cui et al., 2021), translating these successes to the field remains difficult. Key obstacles include ensuring the bioavailability of pollutants and the survival of non-native engineered species in complex natural environments (Nandy et al., 2021). Future success requires moving beyond pure cultures toward metagenomic approaches and multidisciplinary tools, such as remote sensing, to monitor large-scale implementations.

Synthetic biology is transforming this landscape by shifting from simple pollutant removal to an integrated “Waste-to-Wealth” paradigm. Technologies like CRISPR-Cas9 and metabolic engineering now allow for the creation of microbial factories that convert agricultural residues and industrial waste into biofuels, biomaterials, and high-value natural products (Hassard et al., 2024; Jiang et al., 2025). This approach recognizes waste as a resource rather than a burden (Luisi et al., 2014), promoting economic sustainability by minimizing pollution while generating revenue from biomanufacturing. However, the environmental release of these organisms raises substantial biosafety concerns. Stakeholder analysis highlights fears regarding unintended gene transfer and containment failure (Simon et al., 2025). Because traditional biocontainment can be circumvented by evolutionary pressure, advanced strategies such as synthetic protein design and metabolic dependence on non-standard amino acids are being developed to prevent “evolutionary escape” (Mandell et al., 2015). Furthermore, engineered strains may experience genetic instability, metabolic burden, and reduced fitness under environmental conditions, potentially affecting their long-term performance and reliability (Lau et al., 2015). Risk assessment should also consider the intended deployment strategy, as closed photobioreactors and immobilized systems generally present lower ecological risks than open-environment applications. Establishing standardized risk assessment protocols and transparent public engagement is essential to balance such innovations with ecological safety (Calatrava et al., 2024).

The practical transition toward this paradigm is already evidenced by several bioengineered systems operating at pilot and commercial scales. A prominent example is the Algal Turf Scrubber (ATS) technology bioengineered systems that utilize naturally seeded filamentous algae on sloping surfaces to treat wastewater while simultaneously producing biofuel feedstock (Adey et al., 2011). These systems demonstrate exceptional productivity, often 5–10 times higher than traditional land-based agriculture, with routine processing volumes reaching 40–80 million liters per day (Adey and Bannon, 2011). Field studies, such as those conducted on the Great Wicomico River, have shown that utilizing three-dimensional substrates can enhance productivity to 47.7 g·m^-^²·d^-^¹, yielding biomass capable of generating ethanol at rates significantly higher than conventional corn-based agriculture (Adey et al., 2013).

Complementing ATS, High-Rate Algal Ponds (HRAP) leverage microalgae-bacteria consortia for intensive nutrient recovery from municipal wastewater. Research indicates that HRAPs can achieve nitrogen removal efficiencies of 51.8 - 90.2% and phosphorus removal of 40–100%, depending on climatic and operational conditions (Delgadillo-Mirquez et al., 2016; Zhou et al., 2006). Performance in these systems exhibits strong diurnal variations, with peak removal rates occurring during maximum photosynthetic activity in daylight hours (Picot et al., 1993). While strains such as *Chlorella*, *Arthrospira* (Spirulina), and *Scenedesmus* can achieve nutrient removal rates of up to 90%, significant challenges remain regarding commercial scalability. Current production costs range from 1.98 to 9.69 EUR/kg, and land requirements pose economic bottlenecks for widespread adoption (Dias et al., 2025; Acién et al., 2016). The economic feasibility of this model is bolstered by the recovery of valuable byproducts during treatment. Cyanobacteria can achieve up to 100% organic matter removal and significant nutrient recovery (53% Nitrogen, 88% Phosphorus) while fixing CO_2_ at impressive rates of 3.54–4.2 g/L (Shahid et al., 2021). For instance, *Spirulina platensis* grown in aquaculture wastewater produces valuable phycocyanin pigments while sequestering carbon, matching the yields of synthetic media (Kumari et al., 2023). Furthermore, phyconanotechnology approaches allow for the recovery and valorization of captured heavy metals into functional nanoparticles (Ciani and Adessi, 2023).

Beyond aquatic remediation, cyanobacteria are being deployed for dryland restoration through the creation of biological soil crusts (biocrusts). Inoculating degraded soils with native nitrogen-fixing species, particularly *Nostoc commune*, has been shown to increase soil surface coverage by up to 50% and significantly boost organic carbon and nitrogen content within three months (Román et al., 2018; Roncero-Ramos et al., 2019). Recent advances have integrated these biological agents with soil-fixing chemicals and superabsorbent polymers to rapidly improve soil aggregate stability and reduce erodibility (Park et al., 2017). These diverse applications demonstrate that cyanobacterial inoculation is no longer just a laboratory concept but a viable biotechnological approach for combating desertification and managing large-scale wastewater streams.

In conclusion, engineered cyanobacteria represent a versatile and sustainable “living factory” platform. By integrating smart genetic circuits, SMC, and advanced immobilization techniques, these systems address the dual challenges of environmental pollution and resource scarcity. However, significant challenges remain, including the economic costs associated with large-scale cultivation, photobioreactor infrastructure, biomass harvesting, and downstream processing. Ultimately, this light-driven biological process aligns directly with the United Nations Sustainable Development Goals (SDGs), offering a path toward clean water, renewable energy, and a resilient circular bioeconomy (de Lorenzo et al., 2018).

## 6 Conclusion

The transition of cyanobacteria from simple photosynthetic organisms to sophisticated, bioengineered platforms marks a new era in environmental management. As explored in this work, the potential of cyanobacteria extends far beyond carbon sequestration; they represent a modular, “living filter” technology capable of addressing the multifaceted crisis of human-generated waste. By integrating solar energy capture with active detoxification and resource recovery, these organisms offer a carbon-neutral alternative to energy-intensive chemical remediation. The bioengineering roadmap outlined here ranging from the reclamation of toxic heavy metals to the entrapment of microplastics and the enzymatic degradation of synthetic pesticides demonstrates that cyanobacteria can be programmed to handle the most recalcitrant pollutants. Recent advances in living materials, engineered biosensors, and metabolic engineering approaches highlight the potential of these biological systems to move beyond laboratory-scale proof-of-concepts toward field-applicable remediation strategies, although further validation under realistic environmental conditions is required. However, the future of cyanoremediation depends on the responsible deployment of these systems. The integration of advanced biocontainment strategies with high-efficiency immobilization matrices may improve the stability and environmental safety of engineered strains. Nevertheless, several challenges remain, including field-scale validation, process scalability, biosafety considerations, biomass management, regulatory approval, and economic feasibility. Applications such as wastewater treatment and nutrient recovery are approaching practical implementation, whereas engineered strains, living materials, and advanced synthetic biology approaches still require extensive pilot-scale validation and long-term biosafety assessment. Addressing these limitations will be essential for translating promising laboratory findings into reliable and environmentally responsible remediation technologies. Ultimately, by coupling pollutant capture with circular recovery workflows, cyanobacterial platforms provide a sustainable pathway toward a circular bioeconomy. They represent a promising component of next-generation biotechnology, where solar energy is harnessed not only to sustain life but also to restore and protect aquatic and terrestrial ecosystems.
